# Wheel-AINS: A Vehicle Autonomous Positioning System Based on a Wheel-Mounted MIMU Array

**DOI:** 10.3390/mi17070767

**Published:** 2026-06-24

**Authors:** Guangmin Yuan, Guoyuan He, Xiangyang Guo, Ruijie Li, Chenyang Jiao, Xiaoying Li

**Affiliations:** 1 MOE Key Laboratory of Micro and Nano Systems for Aerospace, Northwestern Polytechnical University, Xi’an 710072, China; lrj@mail.nwpu.edu.cn (R.L.); jcyaixuexi@mail.nwpu.edu.cn (C.J.); xiaoy@nwpu.edu.cn (X.L.); 2 School of Marine Science and Technology, Northwestern Polytechnical University, Xi’an 710072, China; hgy1104@mail.nwpu.edu.cn; 3 School of Automation, Northwestern Polytechnical University, Xi’an 710072, China; xiangyangguo@mail.nwpu.edu.cn

**Keywords:** vehicle-mounted inertial navigation system, wheel rotation modulation, MEMS-IMU array, Kalman filtering, differential fusion

## Abstract

In satellite-denied environments such as urban canyons, tunnels, and underground parking facilities, achieving high-precision autonomous positioning for vehicles remains a critical challenge. Although high-precision inertial measurement units (IMUs) can provide accurate dead reckoning, their deployment is limited by cost, size, and power consumption, making low-cost, microelectromechanical systems IMUs (MIMUs) an attractive alternative solution. However, the single MIMU suffers from substantial measurement noise and bias instability, leading to rapid error divergence that cannot sustain long-term autonomous navigation. To address the above issues, this paper proposes an autonomous positioning system based on a wheel-mounted MIMU array (Wheel-AINS). The system adopts a differential layout in which multiple low-cost MIMU chips are installed at the center of each of the left and right rear wheels, forming redundant sensor arrays. By differentially fusing symmetrically mounted chips, common-mode noise and zero bias are effectively canceled while the wheel rotation provides natural rotational modulation. The fused gyroscope outputs and known wheel radius are then used to estimate the vehicle forward speed, replacing traditional odometers. The estimated wheel speed and vehicle kinematic constraints are then integrated within a Kalman filter framework to suppress the error divergence of the inertial navigation system. A dedicated embedded hardware prototype with multi-chip synchronous acquisition and wireless transmission was developed. Three groups of urban road tests with total distances of 0.85 km, 2.14 km, and 2.49 km were conducted. The results indicate that the average position drift rate of the Wheel-AINS is 0.50%, and the average heading RMSE is 12.2°. The closure error of the 2.49 km trajectory is 10.43 m, reduced by approximately 80% compared with a single MIMU. The ablation experiment reveals that the MIMU array fusion module is the primary source of accuracy improvement, reducing the position RMSE from 155.0 m to 10.1 m, while the dual-wheel distance constraint further optimizes the position RMSE to 8.2 m, but increases the heading RMSE from 13.3° to 13.6°. This demonstrates that the proposed method can substantially improve autonomous positioning accuracy while maintaining a notably low system cost, providing a viable technical pathway for long-endurance vehicle navigation in satellite-denied environments.

## 1. Introduction

Precise positioning is the core prerequisite for wheeled vehicles to achieve autonomous navigation and environmental perception [1,2]. Currently, global navigation satellite systems can provide centimeter-level positioning results in open environments [3], but in complex environments such as urban canyons, tunnels, and underground parking lots, multipath effects and signal blockage lead to a significant decrease in accuracy or even complete failure [4,5]. Therefore, developing autonomous relative positioning methods that do not rely on external signals has significant engineering value for improving the robustness and task reliability of vehicle navigation systems.

The inertial navigation system, as a fully autonomous positioning method, has significant advantages in terms of anti-interference capability. With the rapid development of microelectromechanical system (MEMS) technology, MEMS IMUs have been widely applied in mobile robot navigation and pedestrian trajectory estimation due to their small size, low cost, and low power consumption [6,7]. However, due to the large measurement noise and zero bias instability of MEMS sensors, the positioning error of pure inertial navigation accumulates rapidly over time and is difficult to independently meet the requirements of long-term autonomous positioning [8,9]. Therefore, external auxiliary information must be introduced to suppress the error divergence of INSs.

The wheel odometer, as one of the most common sensors for wheeled vehicles, can provide information on the vehicle’s speed or mileage, and has become an effective means to suppress the drift of INS errors [10,11]. Studies have shown that combining the odometer with an INS and vehicle incomplete constraints can significantly suppress positioning and attitude errors and improve the stability of the system [12,13]. However, the measurement accuracy of traditional odometers is significantly affected by road conditions and tire slippage [14], and problems such as data synchronization, hardware integration, and acquisition of reliability information among different systems also restrict the practical application of the ODO/INS scheme [15].

To address these issues, Niu et al. proposed a trajectory estimation system based on a wheel-mounted MEMS IMU—Wheel-INS [16]. They installed a MEMS IMU at the center of the non-steering wheel and used the gyroscope output to calculate the wheel speed by combining it with the wheel radius, thereby replacing the traditional odometer. At the same time, by leveraging the rotational modulation effect generated by the periodic rotation of the wheel, the constant zero bias of the sensor perpendicular to the rotation axis is converted into a sine signal, which is then integrated to cancel each other out [17]. Experiments show that the maximum horizontal position drift rate of Wheel-INS is less than 1.8%, and the positioning accuracy is improved by approximately 23% compared to the traditional ODO/INS scheme [16]. The idea of installing the IMU on the wheel to achieve trajectory estimation was first proposed by Collin et al. from the University of Tampere, Finland, in 2015 [15,18], but their scheme relied on the assumption of uniform motion and did not fully consider the installation deviation between the IMU and the wheel center. In subsequent studies, Wu et al. further proposed the Wheel-INS2 system, exploring configuration schemes of installing multiple IMUs at different positions on the wheel and the vehicle body, including a dual-wheel IMU configuration, one-wheel IMU plus one-vehicle body IMU configuration, and dual-wheel IMU plus one-vehicle body IMU configuration. Experimental results show that the multi-IMU configuration outperforms the single Wheel-INS scheme in terms of positioning and heading accuracy, with the position drift rate of the one-wheel IMU plus one-vehicle body IMU configuration being the lowest, at 0.69% [19].

MEMS-based inertial navigation systems (INSs) face critical challenges in balancing cost and performance. While rotation modulation techniques effectively suppress constant sensor biases through periodic IMU rotation [13], they remain vulnerable to random noise and high-frequency vibrations. Conversely, MIMU arrays demonstrate superior noise suppression via spatial redundancy [7], yet fail to address deterministic errors. This fundamental limitation motivates our investigation into a hybrid domain error suppression architecture that synergizes temporal modulation and spatial filtering.

Although the Wheel-INS scheme shows promising application prospects, its design that utilizes only a single low-precision MEMS IMU has two inherent limitations: Firstly, the measurement noise of a single MEMS IMU is relatively large. Even if some constant deviations are eliminated through rotational modulation, the integral error caused by random noise is still significant [20]. Secondly, rotational modulation can only eliminate the constant deviations perpendicular to the rotation axis, while the constant deviations along the rotation axis cannot be eliminated through rotational modulation and will still lead to the long-term error divergence of the INS [21].

In recent years, MEMS IMU array technology has attracted much attention due to its potential to effectively improve the accuracy of inertial measurement at a low cost. Bayard and Ploen were the first to propose the concept of virtual gyroscopes, by installing multiple gyroscopes of the same accuracy on each axis, and obtaining equivalent accuracy superior to that of a single gyroscope through information fusion [22]. Chang et al. further studied the multi-MIMU system with non-orthogonal redundant structure, designed a conical configuration structure with 4/5/6/8 gyroscopes, and fused the array signals through the optimal Kalman filtering algorithm. Experiments showed that the angle random walk and rate random walk noise of the 4-MIMU system were reduced by approximately 3.5 times and 2.5 times respectively [23]. Niu et al. conducted a systematic dynamic navigation performance evaluation of an array composed of 16 MEMS IMU chips, and found that after precise calibration and compensation for device errors and installation angle errors, the plane position error of the IMU array during 30 s of GNSS failure was reduced by approximately 3.38 times compared to a single IMU, close to the theoretical value √16 = 4 [24]. He et al. in their previous work designed an 8-MIMU array structure with differential distribution, using the positive correlation of the error characteristics of the same batch of MEMS chips, through differential processing to cancel common-mode noise and zero bias, combined with a two-stage Kalman filtering fusion framework, achieving a closure error of 0.36% in a complex path pedestrian navigation test of 1.4 km [25]. Based on this, He et al. further proposed a hybrid domain error suppression architecture, combining the MIMU differential array with single-axis rotation modulation technology to jointly suppress errors in the time domain (periodic rotation) and the space domain (array redundancy), and experiments showed that the navigation error was reduced by one order of magnitude compared to a single MEMS-IMU [26]. Skog et al. proposed a self-calibration method for inertial sensor arrays based on maximum likelihood estimation, which can simultaneously estimate the scale factors, installation deviations, zero bias, and sensor positions of accelerometers and gyroscopes [27].

In the field of vehicle-integrated navigation, vehicle kinematic constraints—especially non-holonomic constraints—have been proven to be an effective method for suppressing the divergence of INS errors [28,29]. The NHC assumes that the lateral and vertical velocities of the vehicle are zero under conditions of no skidding and jumping, and this virtual measurement can effectively constrain the divergence of INS velocity errors [30]. For wheeled vehicles with dual-wheel drive, the fixed spatial relationship between the left and right wheels—that is, the wheelbase—constitutes a rigid body constraint: regardless of the vehicle’s motion state, the relative position vector of the rotation centers of the left and right wheels in the vehicle coordinate system remains unchanged. This dual-wheel distance constraint couples the independently calculated position estimates of the dual-wheel INS into a unified rigid body motion, which can effectively suppress the relative position drift between the wheels caused by the accumulation of sensor errors, thereby indirectly constraining the vehicle’s heading and trajectory divergence [31]. Otegui et al. combined a low-cost IMU installed on the rear wheel with wheel speed information to verify the feasibility of improving positioning accuracy by leveraging the spatial constraints of the left and right wheels [32].

Therefore, this paper proposes a vehicle autonomous positioning system based on wheel-mounted MIMU arrays—Wheel-AINS. This system inherits the basic architecture of Wheel-INS. MIMU arrays are installed on the left and right rear wheels of the vehicle. It fully utilizes the inherent rotational platform characteristics of the wheels, and synergistically combines the two error suppression mechanisms of rotational modulation and array averaging in the time-space dual domain. It also integrates non-holonomic constraints, wheel speed observations, and dual-wheel distance constraints to construct a multi-dimensional redundant observation system. In addition, we developed a physical prototype of a MIMU array specifically for wheel installation, which achieved multi-chip data acquisition and wireless transmission. We also conducted on-road experimental verification in real scenarios, verifying the effectiveness of the proposed method.

Specifically, the main contributions of this paper can be summarized as follows:

(1) A vehicle autonomous positioning method based on the installation of MIMU arrays on wheels was proposed. The distributed differential MIMU array was introduced into the wheel-mounted architecture. Sensor arrays were deployed on the left and right rear wheels of the vehicle. The inherent periodic rotational motion of the wheels formed a rotational modulation effect, which was combined with the array differential fusion technology to construct a time-space dual-domain error suppression architecture.
(2) An information fusion framework integrating multiple kinematic constraints was constructed. The incomplete constraints, wheel speed measurements, and double-wheel distance constraints were all incorporated into a 15-dimensional extended Kalman filter error state. A rigid body constraint equation was established based on the fixed spatial relationship between the left and right wheels, thereby enhancing the observability of the position and heading states.
(3) An embedded wheel-mounted MIMU array prototype with multi-chip synchronous acquisition and wireless transmission was developed, and field vehicle experiments were carried out for verification. The effectiveness of the proposed method was verified on a 2.49 km closed-loop trajectory covering various urban conditions. The closure error after fusion was only 10.43 m (approximately 0.42% of the driving distance), which was an approximately 80% reduction compared with the optimal result of a single MIMU.

The remainder of this paper is organized as follows: Section 2 introduces the hardware structure and coordinate system; Section 3 elaborates on the methodology of Wheel-AINS, including the error state model, the observation model, and the multi-constraint fusion filtering architecture; Section 4 conducts the vehicle positioning experiment; Section 5 summarizes this article.

## 2. System Hardware and Coordinate Transformation

### 2.1. System Hardware

**Figure 1:**
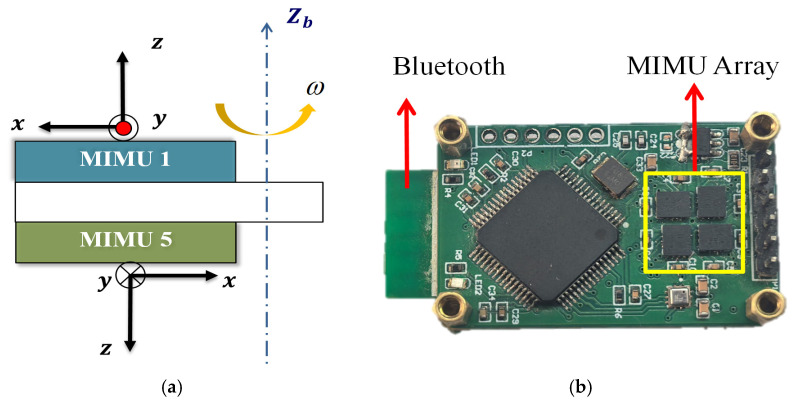
MIMU array’s hardware structure: (**a**) shows the schematic diagram of the differential array structure, and (**b**) is the Wheel-AINS navigation chip we developed.

The hardware of Wheel-AINS is shown in Figure 1. A MIMU array is installed at the wheel center position of each of the left and right rear wheels (non-steering wheels) of the vehicle, with each array consisting of eight MEMS IMU chips. The IMU chips in each array are arranged in a differential layout, with four chips mounted on the front side of the PCB and the remaining four on the back side. The sensitive axes of the front-side chips are opposite to those of the back-side chips. The advantage of this differential layout lies in the fact that the error characteristics of MEMS chips from the same batch exhibit positive correlation. By subtracting the outputs of oppositely mounted chips, common-mode noise and zero bias can be canceled while retaining the true angular velocity/acceleration signals.

Taking the Z-axis of the gyroscope as an example, the measurement outputs of the front chip and the back chip are respectively:

(1) $$\left\{\begin{array}{l} {\tilde{\omega }}_{\mathrm{front}}=\omega +b_{\mathrm{front}}+n_{\mathrm{front}}\\ {\tilde{\omega }}_{\mathrm{back}}=-\omega +b_{\mathrm{back}}+n_{\mathrm{back}} \end{array}\right.$$

where ω represents the true value of the angular velocity of the carrier rotating around the Z-axis of the gyroscope; b represents the zero bias of the gyroscope chip itself, which is a typical slow-time-varying drift error of an inertial device; and n represents the random Gaussian white noise of the gyroscope angular velocity measurement signal.

After differential processing, the equivalent measurement value ${\tilde{\omega }}_{\mathrm{diff}}$ is defined:

(2) $${\tilde{\omega }}_{\mathrm{diff}}=\frac{{\tilde{\omega }}_{\mathrm{front}}-{\tilde{\omega }}_{\mathrm{back}}}{2}=\omega +\frac{b_{\mathrm{front}}-b_{\mathrm{back}}}{2}+\frac{n_{\mathrm{front}}-n_{\mathrm{back}}}{2}$$

Due to the positive correlation of zero bias within the same batch of chips, the residual zero bias after differential processing will be significantly reduced. This differential structure has been verified in our previous work [25].

### 2.2. Coordinate System Definition

The Wheel-AINS system involves four coordinate frames to support strapdown inertial calculation, multi-sensor data fusion and vehicle kinematic constraint construction. The definitions of each frame and their transformation relationships are specified as follows, which follow the standard conventions of the inertial navigation field.

(1) Navigation frame (*n*-frame)

The navigation frame adopts the standard north-east-down (NED) right-handed orthogonal coordinate system. Its origin is located at the projection of the vehicle’s center of mass on the local horizontal plane at the initial moment. The $x_{n}$ axis points to the geographic north, the $y_{n}$ axis points to the geographic east, and the $z_{n}$ axis points vertically downward along the local plumb line, aligned with the gravity direction. This frame serves as the global reference for all position, velocity and attitude outputs of the navigation system.

(2) Vehicle body frame (*v*-frame)

The vehicle body frame is a right-handed orthogonal coordinate system rigidly fixed to the vehicle chassis, with its origin coinciding with the vehicle’s center of mass. The $x_{v}$ axis points forward along the longitudinal axis of the vehicle body, the $y_{v}$ axis points rightward along the lateral axis, and the $z_{v}$ axis points vertically downward perpendicular to the vehicle chassis plane.

(3) Wheel body frame (*w*-frame)

The wheel body frame is rigidly fixed to the wheel hub, with its origin coinciding with the geometric center of the wheel. The $x_{w}$ axis is aligned with the wheel rotation axis and points to the right side of the vehicle; the $y_{w}$ axis points forward along the radial direction of the wheel; the $z_{w}$ axis points downward along the radial direction of the wheel, satisfying the right-hand rule.

Since the MIMU arrays are mounted on non-steering rear wheels, the relative attitude between the w-frame and v-frame remains fixed except for the rotation around the $x_{w}$ axis. The wheel rotational angular velocity measured by the gyroscope is the projection along the $x_{w}$ axis, which is the core observation for wheel speed estimation.

(4) MIMU array body frame (*b*-frame)

The MIMU array body frame is fixed to each MIMU chip, with its origin at the measurement center of the inertial sensor. The axes of the *b*-frame are completely consistent with the sensitive axes of the tri-axis gyroscope and tri-axis accelerometer inside the MIMU chip. For the differential layout MIMU array, the sensitive axes of front-side and back-side chips are set opposite, which provides the physical basis for common-mode noise and zero bias cancelation through differential processing.

The transformation relationship between coordinate systems is represented by a rotation matrix $C_{a}^{b}$, which denotes the coordinate transformation from a system to b system.

## 3. Methods

The core objective of MIMU array data fusion is to fuse the redundant measurement information from N low-precision IMU chips into a high-precision measurement output of a single “virtual IMU”. According to the theory of random error, if N sensor signals with comparable measurement error levels and independent of each other are appropriately fused, the measurement error can be reduced by approximately $\sqrt{N}$ times [24]. This paper adopts the differential fusion framework verified in previous work [25].

Each MIMU chip in the MIMU array has been calibrated, and the angular velocity ${\tilde{\omega }}_{k}^{b_{k}}$ and acceleration ${\tilde{f}}_{k}^{b_{k}}$ output by the k MIMU array can be expressed as:

(3) $$\left\{\begin{array}{l} {\tilde{\omega }}_{k}^{b_{k}}=\omega_{k}^{b_{k}}+b_{g,k}+n_{g,k}\\ {\tilde{f}}_{k}^{b_{k}}=f_{k}^{b_{k}}+b_{a,k}+n_{a,k} \end{array}\right.$$

where $\omega_{k}^{b_{k}},f_{k}^{b_{k}}$ is the projection of true angular velocity and acceleration in the *b*-frame; $b_{g,k},b_{a,k}$ represents the zero-bias error; $n_{g,k},n_{a,k}$ represents the Gaussian noise.

The measured values of each MIMU chip are uniformly projected onto the *w*-frame:

(4) $$\left\{\begin{array}{l} {\tilde{\omega }}_{k}^{w}=C_{b_{k}}^{w}{\tilde{\omega }}_{k}^{b_{k}}\\ {\tilde{f}}_{k}^{w}=C_{b_{k}}^{w}{\tilde{f}}_{k}^{b_{k}} \end{array}\right.$$

**Figure 2:**
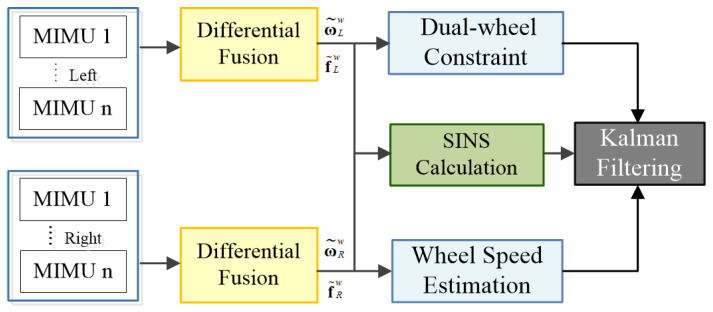
The algorithmic process of the Wheel-AINS.

Let the MIMU array of the left and right wheels be denoted as L and R respectively, and their measured values in the *w*-frame are represented as ${\tilde{\omega }}_{L}^{w},{\tilde{f}}_{L}^{w}$ and ${\tilde{\omega }}_{R}^{w},{\tilde{f}}_{R}^{w}$, respectively. The calculation process of the algorithm is shown in Figure 2.

### 3.1. Estimation of Wheel Speed

Using the gyroscope output of the virtual MIMU and the wheel radius, the forward speed of the wheel can be estimated. For a single wheel, the rotational angular rate around the $x_{w}$ axis in the *w*-frame w is the measurement value of the gyroscope’s X-axis. Assuming the effective radius of the wheel is r, the forward speed $\tilde{v}$ at the center of the wheel can be estimated as:

(5) $$\tilde{v}=r\cdot {\tilde{\omega }}_{x}^{w}$$

where ${\tilde{\omega }}_{x}^{w}$ represents the X-axis output of the gyroscope in the MIMU array.

For the left and right wheels, we obtain the left forward speed ${\tilde{v}}_{L}$ and the right forward speed ${\tilde{v}}_{R}$, respectively. According to the dual-wheel differential kinematics model [31,32], the vehicle forward speed ${\tilde{v}}_{x}^{v}$ can be estimated as follows:

(6) $${\tilde{v}}_{x}^{v}=\frac{{\tilde{v}}_{L}+{\tilde{v}}_{R}}{2}$$

The observed forward velocity of the vehicle can directly replace the traditional odometer.

It should be noted that single-axis rotational modulation can only modulate constant bias perpendicular to the rotation axis direction. The zero bias of the gyroscope in the rotation axis direction cannot be suppressed through rotational modulation. To address this issue, Wheel-AINS employs two mechanisms: firstly, during vehicle static periods, static IMU data is collected, and the mean value of the gyroscope X-axis output is estimated and compensated for its initial zero bias; secondly, using the differential processing of the chips on the front and back sides of the array (Equation (2)), further residual zero bias with positive correlation in the same batch of chips is suppressed.

### 3.2. Error State Model

Wheel-AINS employs an error state Kalman filter for information fusion. Ignoring the rotation of the Earth and changes in the *n*-frame (applicable to short-term autonomous positioning for low-cost MEMS), the simplified differential equation for the error state is:

(7) $$\left\{\begin{array}{l} \delta {\dot{r}}^{n}=\delta v^{n}\\ \delta {\dot{v}}^{n}=-\phi \times f^{n}+C_{b}^{n}\delta f^{b}\\ \dot{\phi }=-C_{b}^{n}\delta \omega^{b} \end{array}\right.$$

where $\delta r^{n},\delta v^{n}$ and ϕ represent position error, velocity error, and attitude error (misalignment angle) respectively; $f^{n}$ denotes the specific force vector in the navigation system; $\delta \omega^{b}$ and $\delta f^{b}$ represent the measurement errors of the gyroscope and accelerometer respectively, which can be modeled as follows:

(8) $$\left\{\begin{array}{l} \delta \omega^{b}=b_{g}+n_{g}\\ \delta f^{b}=b_{a}+n_{a} \end{array}\right.$$

where $b_{g}$ and $b_{a}$ are zero-bias errors, $n_{g}$ and $n_{a}$ are measurement Gaussian noises.

Taking into account both the observability and computational efficiency of the system, a 15-dimensional error state vector x(t) is defined as follows:

(9) $$x(t)={\left[{\left(\delta r^{n}\right)}^{T}\;{\left(\delta v^{n}\right)}^{T}\;\phi^{T}\;b_{g}^{T}\;b_{a}^{T}\;\right]}^{T}$$

The state transition equation is:

(10) $$\dot{x}(t)=F(t)x(t)+G(t)w(t)$$

where F(t) represents the system transfer matrix; G(t) represents the noise distribution matrix, which describes the driving effect of random noise on the rate of change in each error state; w(t) represents the system process noise vector, which is composed of random measurement noise from the inertial sensors.

### 3.3. Observation Model

The observation model of Wheel-AINS incorporates three types of constraint information: non-holonomic constraints (NHC), wheel speed observations, and dual-wheel distance constraint. These three types of observations collaboratively work together in measurement updates to jointly constrain the error divergence of the INS.

#### 3.3.1. Non-Holonomic Constraints

Under normal driving conditions without sideslip or jump, the lateral velocity $v_{y}^{v}$ and vertical velocity $v_{z}^{v}$ of the vehicle in the *v*-frame should be close to zero [28,29]. In the *n*-frame, the vehicle speed calculated by the inertial navigation system is ${\hat{v}}^{n}$. The conversion relationship between ${\hat{v}}^{n}$ and ${\hat{v}}^{v}$ is as follows:

(11) $${\hat{v}}^{v}=C_{n}^{v}{\hat{v}}^{n}={\left[{\hat{v}}_{x}^{v},{\hat{v}}_{y}^{v},{\hat{v}}_{z}^{v}\right]}^{T}$$

The NHC observation residual vector $z_{NHC}$ is defined as:

(12) $$z_{NHC}=\left[\begin{array}{c} {\hat{v}}_{y}^{v}\\ {\hat{v}}_{z}^{v} \end{array}\right]-\left[\begin{array}{c} 0\\ 0 \end{array}\right]=H_{NHC}x+n_{NHC}$$

where $H_{NHC}$ is the corresponding observation matrix, which establishes a linear mapping between residuals and error states, and $n_{NHC}$ is the observation noise.

#### 3.3.2. Wheel Speed Observation

Under the error state framework, the velocity observation residual $z_{v}$ can be written as:

(13) $$z_{v}={\hat{v}}_{x}^{v}-{\tilde{v}}_{x}^{v}=H_{v}x+n_{v}$$

where ${\hat{v}}_{x}^{v}$ is the vehicle speed calculated by the inertial navigation system, ${\tilde{v}}_{x}^{v}$ is the vehicle speed estimated by wheel speed, $H_{v}$ is the observation vector, and $n_{v}$ is the observation noise.

#### 3.3.3. Dual-Wheel Constraint

The fixed spatial relationship between the left and right wheels provides additional constraint information. Let the position difference in the left and right wheels in the *v*-frame be the fixed baseline vector $d_{LR}^{v}={\left[0,d,0\right]}^{T}$, where d represents the track width between the left and right wheels. Then the dual-wheel constraint observation residual $z_{LR}$ between the wheels is:

(14) $$z_{LR}=\left({\hat{r}}_{L}^{n}-{\hat{r}}_{R}^{n}\right)-C_{v}^{n}d_{LR}^{v}=H_{LR}x+n_{LR}$$

where ${\hat{r}}_{L}^{n},{\hat{r}}_{R}^{n}$ is the position estimate value obtained through inertial navigation calculation, $H_{LR}$ is the dual-wheel constraint observation matrix, $n_{LR}$ is the observation noise.

Combine the above three types of observations into a unified observation equation:

(15) $$z=\left[\begin{array}{c} z_{NHC}\\ z_{v}\\ z_{LR} \end{array}\right]=Hx+n=\left[\begin{array}{l} H_{NHC}\\ H_{v}\\ H_{LR} \end{array}\right]x+\left[\begin{array}{l} n_{NHC}\\ n_{v}\\ n_{LR} \end{array}\right]$$

The Kalman update is performed as:

(16) $$\left\{\begin{array}{l} K_{k}=P_{k|k-1}H^{T}{\left(HP_{k|k-1}H^{T}+R\right)}^{-1}\\ x_{k}=x_{k|k-1}+K_{k}(z_{k}-Hx_{k|k-1})\\ P_{k}=(I-K_{k}H)P_{k|k-1} \end{array}\right.$$

After the measurement update, the estimated errors are fed back to correct the navigation states (position, velocity, attitude) and compensate the MIMU sensor biases.

### 3.4. Synergistic Effect of Rotary Modulation and Array

The core innovation of Wheel-AINS lies in the organic combination of two error suppression mechanisms: rotational modulation (in the time domain) and differential fusion of MIMU array (in the spatial domain), forming a dual-domain collaborative error suppression architecture. This architecture has been verified in our previous work [26]. The mechanism analysis is as follows. From the frequency domain perspective, rotational modulation mainly acts on low-frequency errors (constant zero bias), converting constant deviations into periodic signals through periodic rotation, and then integrating them to cancel each other out [17,33]. Array averaging mainly suppresses high-frequency random noise, reducing the noise power spectral density through the statistical averaging of multiple sensors [24]. The combination of the two forms a complementary error suppression architecture of “low-pass” and “high-pass” in the frequency domain.

Specifically, for the constant zero bias $b_{g,k}$ of the *k*th gyroscope, after rotation modulation, the equivalent error in the *n*-frame is:

(17) $$\epsilon^{n}=C_{w}^{n}C_{b}^{w}b_{g,k}$$

During the period $T_{c}$ when the wheel rotates once, the zero-offset component $\epsilon_{⊥}^{n}$ perpendicular to the rotation axis is modulated into a sine wave form in the *n*-frame, and its integral is zero:

(18) $$\int_{0}^{T_{c}}\epsilon_{⊥}^{n}dt\approx 0$$

## 4. Vehicle Positioning Experiment and Result Analysis

To verify the engineering feasibility and positioning accuracy of the vehicle autonomous positioning method proposed in this paper based on the wheel-mounted MIMU array, we conducted autonomous positioning experiments on a real vehicle.

**Figure 3:**
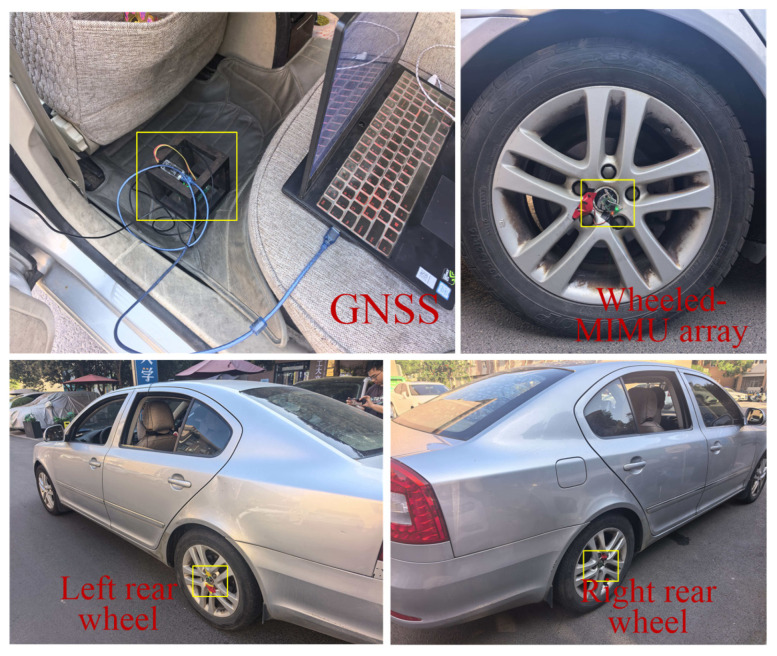
Installation of the wheeled MIMU array for vehicle experiments.

The hardware deployment and installation scheme of the wheel-mounted MIMU array is shown in Figure 3. The array is divided into two groups, left and right, and is rigidly installed at the center positions of the left and right non-steering rear wheel hubs of the experimental vehicle. Each set of MIMUs is powered by an independent re-chargeable battery module, and at the same time, through a Bluetooth communication module, the raw three-axis angular velocity and three-axis force measurement data sampled at high frequency are uploaded in real time to the on-board upper computer for data fusion and navigation calculation. To obtain the true value benchmark for the navigation solution, the experimental vehicle was equipped with a GNSS navigation system, which can provide reference true values. In this experiment, urban built-up area roads were selected as the test scenario, covering typical urban conditions such as multiple intersections, continuous turns, and long straight roads. There are three closed round-trip test routes. The total driving distances of the vehicles on each route are 2.49 km, 2.14 km, and 0.85 km respectively. The MIMU sensor used in the experiment was the BMI160, with a sampling frequency of 100 Hz. The gyroscope has a measurement range of ±2000 deg/s, and a bias instability of approximately 10 deg/h; the accelerometer has a measurement range of ±16 g. The wheel radius is 0.307 m. Due to our hardware installation on the outside of the wheels, we used a body width of 1.769 m as the distance between the two wheels. The measurement noise was set as follows: $\sigma_{NHC}\;=0.0001$ m/s for the non-holonomic constraints, $\sigma_{v}\;=0.0001$ m/s for the wheel speed observation, and $\sigma_{LR}\;=0.2$ m for the dual-wheel distance constraint.

In our experiment, the initial roll and pitch angles were obtained from the accelerometer measurements during the stationary period before the vehicle started moving, using the standard leveling procedure. Since the MIMU used in this study is a consumer-grade device and cannot measure the Earth’s rotational angular velocity, it is impossible to perform the initial alignment of the heading angle without GNSS. The focus of this paper is on the positioning performance of the Wheel-AINS system during GNSS signal loss, so the initial heading is provided by the reference GNSS navigation system at the starting point.

### 4.1. Long-Distance Test

**Figure 4:**
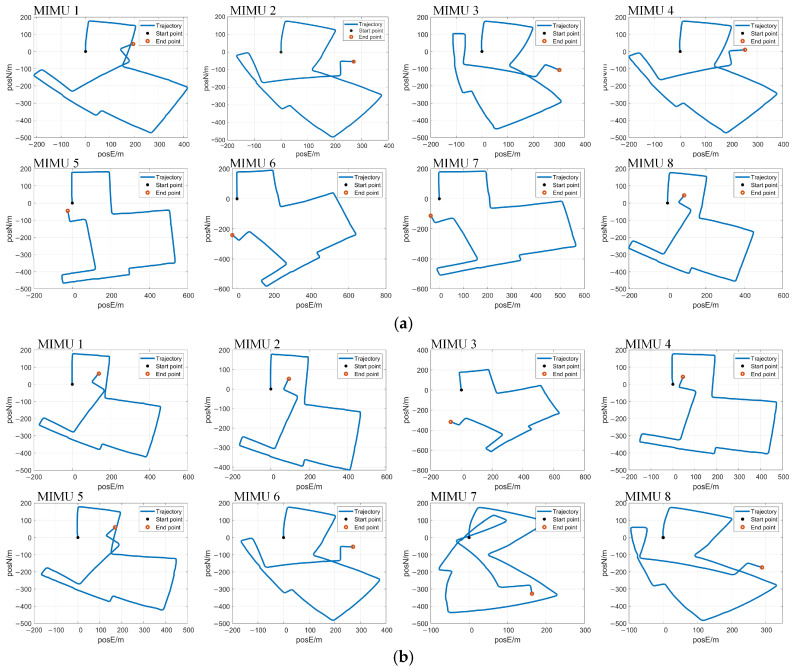
The single MIMU output trajectory results. (**a**) Single MIMU trajectory of the left wheel, (**b**) single MIMU trajectory of the right wheel.

**Figure 5:**
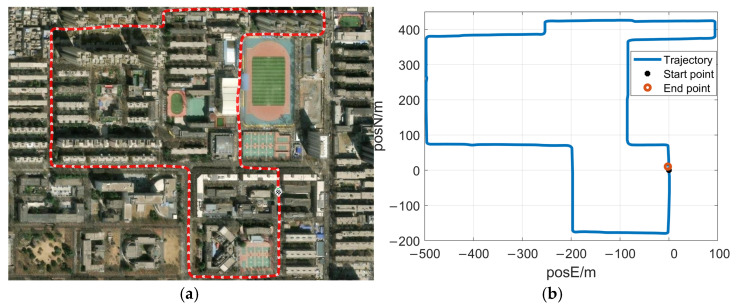
The trajectories of the vehicle positioning experiment: (**a**) represents the satellite positioning result, while (**b**) represents the positioning result of Wheel-AINS.

The trajectory output by the inertial navigation calculation of each independent MIMU in the left-wheel-mounted MIMU array is shown in Figure 4a, and the corresponding output results of each independent inertial measurement unit in the right-wheel-mounted MIMU array are shown in Figure 4b. From the test results, it can be seen that all independent MIMUs in the array have different degrees of closure errors, mainly due to the accumulation of heading errors. The test results of the proposed Wheel-AINS in this paper are shown in Figure 5; it can be seen that the closure error has been significantly reduced.

To verify the effectiveness of the proposed wheel-mounted MIMU array positioning method, this experiment quantitatively compared the key performance indicators of the eight independent MIMUs in the left and right wheel-mounted arrays and the fused Wheel-AINS system. The results are shown in Table 1. The calculated driving distances of all individual MIMUs were highly consistent with the actual distance (2.49 km), with relative deviations all less than 1.2%. This verified the reliability of the wheel-mounted MIMU in estimating vehicle mileage through gyroscope measurement of wheel speed; however, due to the individual performance fluctuations of low-cost MEMS sensors, the closure errors showed significant differences. The closure error distribution of the left wheel array was 51.04–320.7 m, and that of the right wheel array was 63.1–365.8 m. The positioning drift level fluctuated greatly. The closure error of the Wheel-AINS system was only 10.43 m, which improved the accuracy by more than 80% compared to the optimal result of a single MIMU. This suppressed the positioning drift in long-distance position estimation, proving the advantage of the proposed array fusion scheme in improving the accuracy of vehicle autonomous positioning.

**Figure 6:**
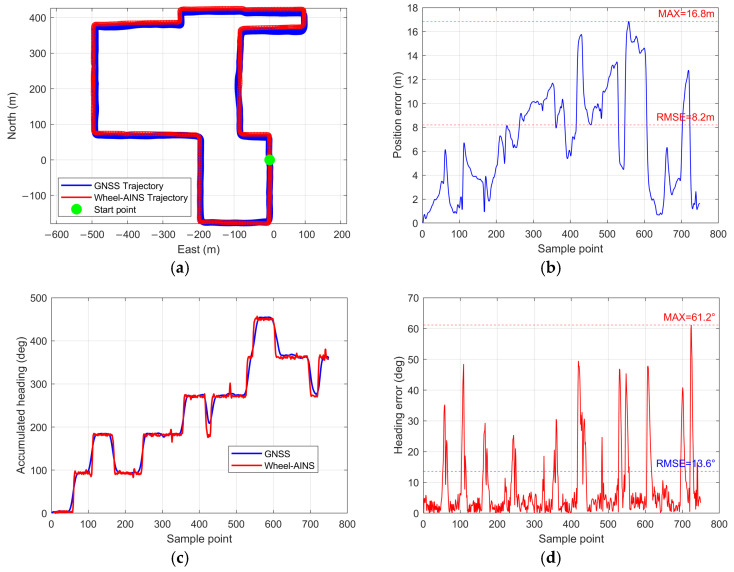
The full-trajectory error evaluation of long-distance test: (**a**) represents GNSS and Wheel-AINS positioning trajectories, (**b**) represents the position error curve of Wheel-AINS, (**c**) represents the accumulative heading, and (**d**) represents the heading error curve of Wheel-AINS.

To accurately quantify the navigation performance of the proposed Wheel-AINS system, a systematic quantitative evaluation of full-trajectory position and heading errors is conducted based on long-distance on-road vehicle test data, and the evaluation results are depicted in Figure 6.

Figure 6a presents the comparison of positioning trajectories, where the blue curve denotes the GNSS reference ground truth trajectory, the red curve corresponds to the trajectory estimated by the Wheel-AINS system, and the green dot marks the trajectory starting point. Limited by the data update rate of approximately 1 Hz of the GNSS receiver employed in the experiment, error comparison is performed exclusively at sampling points with valid GNSS measurements.

Figure 6b plots the position error of the Wheel-AINS system as a function of sampling points, and the error generally exhibits a cumulative fluctuation pattern. Statistical results demonstrate that the root mean square error (RMSE) of the horizontal position over the entire trajectory is 8.2 m, with a maximum position error of 16.8 m. Corresponding to the total travel distance of 2.49 km in this test, the maximum position drift rate is approximately 0.68%.

Figure 6c illustrates the comparison of accumulated heading angles between the GNSS reference and the Wheel-AINS system, where the heading ground truth is calculated via the coordinate difference in adjacent GNSS position points.

Figure 6d shows the heading error curve of the Wheel-AINS system. According to the statistical results, the full-trajectory heading RMSE is 13.6°, and the maximum heading error reaches 61.2°.

### 4.2. Medium-Distance Test

**Figure 7:**
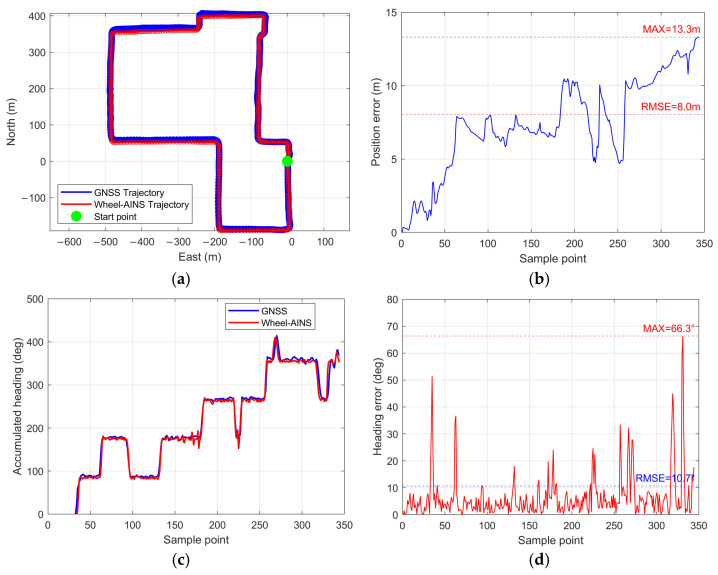
The full-trajectory error evaluation of the medium-distance test: (**a**) represents GNSS and Wheel-AINS positioning trajectories, (**b**) represents the position error curve of Wheel-AINS, (**c**) represents the accumulative heading, and (**d**) represents the heading error curve of Wheel-AINS.

Figure 7 presents the quantitative evaluation results of the full-trajectory navigation performance of the Wheel-AINS in a medium-distance urban road field test. The total mileage of the test is 2.14 km.

Specifically, Figure 7a shows the comparison of positioning trajectories, where the blue curve denotes the GNSS reference ground truth trajectory and the red curve represents the trajectory calculated by the Wheel-AINS system. Figure 7b depicts the variation in the horizontal position error of the Wheel-AINS system with sampling points; the error generally exhibits a gentle accumulation trend. Statistics indicate that the root mean square error (RMSE) of the position over the entire trajectory is 8.0 m, and the maximum position error reaches 13.3 m. Corresponding to the total travel distance of approximately 2.14 km in this test, the position drift rate of the system is better than 0.63%, which validates the effective suppression capability of the wheel-mounted MIMU array fusion scheme on positioning errors. Figure 7c illustrates the comparison curves of accumulated heading angles between GNSS and the Wheel-AINS system. The variation trends of the two curves are relatively consistent in the early stage, while noticeable steering errors emerge in the later stage after multiple steering maneuvers, which conforms to the heading error accumulation characteristic of inertial navigation systems. Figure 7d shows the variation in the heading error of the Wheel-AINS system with sampling points, where the error peaks mainly occur during vehicle steering conditions. According to statistics, the RMSE of the heading over the entire trajectory is 10.7°, and the maximum heading error is 66.3°.

### 4.3. Short-Distance Test

**Figure 8:**
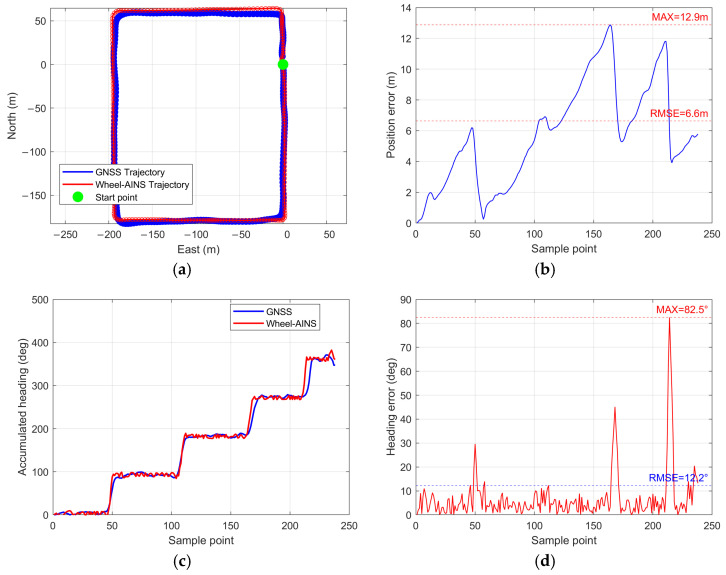
The full-trajectory error evaluation of the short-distance test: (**a**) represents GNSS and Wheel-AINS positioning trajectories, (**b**) represents the position error curve of Wheel-AINS, (**c**) represents the accumulative heading, and (**d**) represents the heading error curve of Wheel-AINS.

To reduce the randomness of the test, we also conducted a short-distance urban road test with a total distance of 0.85 km, following a standard rectangular path. Figure 8 shows the verification results of the full trajectory navigation performance. Figure 8b presents the curve of the horizontal position error of the Wheel-AINS system with respect to the sampling points. The error shows a fluctuating characteristic of stepwise accumulation overall, and the peaks occur at the end of the long straight road. After statistics, the RMSE of the system’s horizontal position throughout the entire journey is 6.6 m, the maximum position error is 12.9 m, and the maximum position drift rate is approximately 1.6%. Figure 8d shows the heading error curve of the system. After statistics, the root mean square error of the system’s heading throughout the entire journey is 12.2°, and the maximum heading error is 82.5°.

This paper conducted three groups of real-vehicle tests on urban roads, covering a short distance of 0.85 km, a medium distance of 2.14 km, and a long distance of 2.49 km. The quantitative statistical results of each performance indicator are presented in Table 2. Horizontal position error and heading error were selected as the core evaluation metrics, each characterized using the root mean square error (RMSE) and the maximum error (MAX). For position error, the relative drift rate, defined as the error normalized by the total traveled distance, is also reported to eliminate the influence of distance variations on performance comparisons.

Regarding the position error characteristics, the absolute position error of the system exhibits a slight upward trend as the traveled distance increases. The position RMSE values for the three test groups are 6.6 m, 8.0 m, and 8.2 m, with corresponding maximum position errors of 12.9 m, 13.3 m, and 16.8 m. In terms of relative drift rate, however, the RMSE-based relative drift rate decreases with increasing distance, showing no evidence of divergence. For the heading error characteristics, the heading RMSE for the three test groups are 12.2°, 10.7°, and 13.6°, with corresponding maximum heading errors of 82.5°, 66.3°, and 61.2°. The relatively small fluctuation in heading RMSE across different distances reflects the stability of the system’s heading estimation.

The multi-trajectory statistical results show that the mean RMSE relative position drift rate across the three tests is 0.50%, with a standard deviation of 0.21%; the mean maximum relative position drift rate is 0.94%, with a standard deviation of 0.40%. The mean heading RMSE is 12.2°, with a standard deviation of 1.19°; the mean maximum heading error is 70.0°, with a standard deviation of 9.09°. These results indicate that the Wheel-AINS system maintains a certain level of stability across different driving scenarios. In summary, the effectiveness of the proposed method is preliminarily validated. The overall relative position drift rate remains below 1%, providing reliable technical support for continuous vehicle positioning in GNSS-denied environments.

### 4.4. Comparison Test

**Figure 9:**
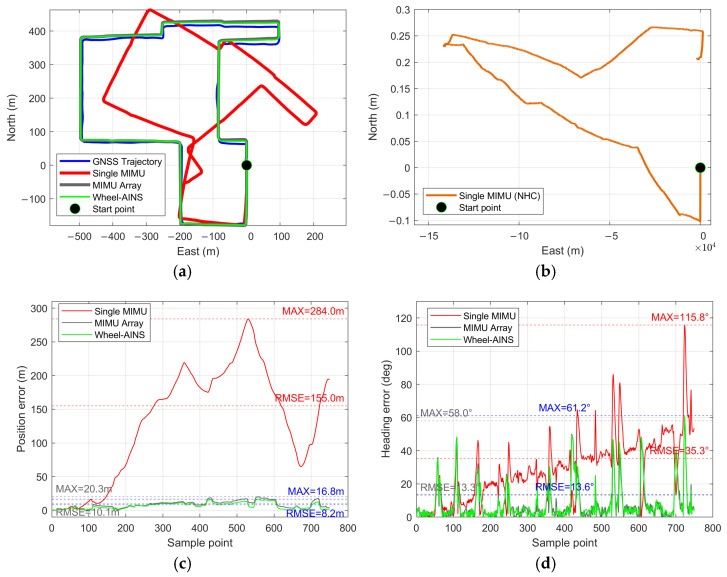
The error results of the comparative test: (**a**) represents the positioning trajectories of the three contrast schemes, (**b**) represents the single MIMU positioning trajectory solely relying on NHC, (**c**) shows the position error of the three comparison schemes, and (**d**) represents the heading error of the three comparison schemes.

We conducted an ablation experiment to evaluate the contribution of each core module. Figure 9 presents the trajectory and error comparison results from the vehicle-mounted positioning ablation study. The objective was to systematically evaluate and quantify the error suppression effect of each core module in the system by hierarchically comparing the navigation performance of three configurations: a single MIMU solution, MIMU array fusion, and the complete Wheel-AINS system. The difference between the MIMU array and the complete Wheel-AINS system lies in whether the dual-wheel distance constraint is employed.

Figure 9a shows a comparison of the positioning trajectories for the three configurations, all of which utilize wheel-speed estimation combined with non-holonomic constraints. The trajectory of the single MIMU configuration diverges significantly with increasing driving distance and number of turns, with its deviation from the reference truth continuously widening. In contrast, the trajectories of both the MIMU array fusion configuration and the Wheel-AINS system show markedly improved consistency with the reference trajectory, remaining significantly closer to the ground truth.

Figure 9b presents the positioning results for a single MIMU using only NHC. It is evident that the position error accumulates dramatically, exceeding 100 km. A comparison with Figure 9a, where a single MIMU is aided by both wheel-speed estimation and NHC, demonstrates that relying solely on the vehicle’s non-holonomic motion constraints is insufficient to suppress the error accumulation of the inertial navigation system.

Figure 9c depicts the evolution of horizontal position errors for three different configurations in Figure 9a. The statistical results indicate that the position error of the single MIMU diverges rapidly during the experiment, yielding a root mean square error (RMSE) of 155.0 m and a maximum position error of 284.0 m over the entire trajectory. Through the multi-sensor redundancy of the MIMU array fusion, random errors are significantly mitigated, reducing the overall position RMSE to 10.1 m with a maximum position error of 20.3 m. This validates the core contribution of the array fusion module to positioning accuracy. The complete Wheel-AINS system, which further incorporates the dual-wheel distance constraint, achieves additional optimization, resulting in a final position RMSE of just 8.2 m and a maximum position error of 16.8 m.

Figure 9d shows the heading error evolution for the three configurations. The heading of the single MIMU configuration is severely affected by gyroscope bias, with the drift continuously increasing during the experiment, resulting in a heading RMSE of 35.3° and a maximum heading error of 115.8°. The MIMU array fusion, using redundant observations from multiple gyroscopes, effectively suppresses this heading drift, reducing the heading RMSE to 13.3° with a maximum heading error of 58.0°. This constitutes the primary source of heading accuracy improvement in the system. The complete Wheel-AINS system maintains excellent heading performance, with a heading RMSE of 13.6° and a maximum heading error of 61.2°, which is comparable to the MIMU array fusion configuration.

In summary, the ablation experiment shows the performance contribution of each module. The MIMU array fusion module serves as the fundamental basis for system accuracy improvement, simultaneously suppressing random errors in both position and heading. The contribution of the dual-wheel constraint module to error suppression is, by comparison, relatively limited. Furthermore, a comparison of the performance of a MIMU array and a single MIMU under rotational modulation can be found in our prior work [26].

To compare our work with the closest existing research, we provide a reference comparison with Wheel-INS2 [19]. However, it should be noted that this study used the consumer grade BMI160 chip, while previous studies used the ICM20602 chip. The best-performing configuration of Wheel-INS2, which places one IMU on a wheel and another on the vehicle body, achieved a position drift rate of 0.69% on a Pioneer 3DX robot platform over a 1.22 km trajectory. In the present study, Wheel-AINS achieves a mean position drift rate of 0.50% and a maximum position drift rate of 0.78%. It must be emphasized, however, that owing to differences in chip specifications, test vehicles, and experimental trajectories, these drift rates do not constitute a directly controlled benchmark comparison. The central question addressed in this study is not whether an array-based wheel-mounted system outperforms a multi-location multi-IMU configuration, but rather whether equipping each wheel with a MIMU array can serve as a viable and effective replacement for the single MIMU on that same wheel. The above results indicate that the drift rates of the two systems reside within the same order of magnitude, providing preliminary validation of the feasibility of the proposed approach.

## 5. Conclusions

This paper presents a vehicle autonomous positioning system based on a wheel-mounted multi-inertial measurement unit (MIMU) array—the Wheel-AINS system. This system expands the single-wheel, single-MIMU scheme to a redundant configuration, with MIMU arrays being equipped on the left and right rear wheels respectively. It integrates multi-dimensional motion information such as non-holonomic constraints, wheel speed observation, and dual-wheel constraints, and constructs a hybrid filtering architecture. Three sets of urban road tests were conducted, with distances of 0.85 km, 2.14 km, and 2.49 km, respectively. The experimental results show that the average position drift rate of the Wheel-AINS is 0.50%, and the single trajectory drift rates are 0.78%, 0.37%, and 0.33%, respectively. The ablation experiment shows that the MIMU array reduced the position RMSE from 155.0 m (single MIMU) to 10.1 m, while the dual-wheel distance constraint further improves the position RMSE to 8.2 m, but increases the heading RMSE from 13.3° to 13.6°. Therefore, the MIMU array fusion module is the main source of system accuracy improvement, while the dual-wheel constraint module provides additional but relatively limited positional accuracy improvement, but with a decrease in heading accuracy. This proposed system is expected to achieve higher autonomous positioning accuracy while maintaining a low cost, providing a technical path for long-term autonomous positioning of wheeled vehicles in environments without satellite navigation.

However, the proposed method still has certain limitations. Due to the limited range of the consumer-grade MEMS gyroscope relied upon by the current system (usually not exceeding ±2000°/s), the applicable vehicle speed is restricted to approximately 40 km/h or below, making it difficult to cover a wider driving scenario such as intercity roads and expressways. Future research will focus on the study of an angular velocity auxiliary estimation method based on the centripetal force signals of the MIMU array. By using the differential output of the chips symmetrically installed in the MIMU array to cancel out the vehicle acceleration and the gravitational component, the centripetal acceleration component generated by the wheel rotation is extracted. Then, through the inverse solution of the centripetal acceleration and angular velocity relationship, the wheel speed is determined, thereby increasing the effective angular velocity range of the system and expanding the application scenarios of Wheel-AINS.

## Figures and Tables

**Table 1:** The results of vehicle-mounted autonomous positioning, comparison of errors between a single MIMU and Wheel-AINS.

Data Source	Closure Error (m)		Mile (m)	
	Left	Right	Left	Right
MIMU 8	97.5	338.2	2477.9	2477.8
MIMU 7	117.9	365.8	2491.8	2488.9
MIMU 6	242.8	276.5	2483.7	2483.4
MIMU 5	51.04	179.8	2501.8	2501.1
MIMU 4	253.5	63.1	2482.7	2480.9
MIMU 3	320.7	325.4	2474.3	2471.7
MIMU 2	242.3	107.8	2485.2	2484.8
MIMU 1	200.2	151.9	2486.1	2482.4
Wheel-AINS	10.43		2494.1	

**Table 2:** The test results of Wheel-AINS under three different paths.

Data Source	Position Error (m)		Heading Error (deg)	
	RMSE	MAX	RMSE	MAX
Long-distance (2.49 km)	8.2 (0.33%)	16.8 (0.68%)	13.6	61.2
Medium-distance (2.14 km)	8.0 (0.38%)	13.3 (0.63%)	10.7	66.3
Short-distance (0.85 km)	6.6 (0.78%)	12.9 (1.5%)	12.2	82.5
Mean	0.50%	0.94%	12.2	70.0
Standard deviation	0.21%	0.40%	1.19	9.09

## Data Availability

The original contributions presented in this study are included in the article. Further inquiries can be directed to the corresponding author.
